# Plasticity of Type I Interferon-Mediated Responses in Cancer Therapy: From Anti-tumor Immunity to Resistance

**DOI:** 10.3389/fonc.2018.00322

**Published:** 2018-08-21

**Authors:** Megha Budhwani, Roberta Mazzieri, Riccardo Dolcetti

**Affiliations:** The University of Queensland Diamantina Institute, Translational Research Institute, Brisbane, QLD, Australia

**Keywords:** interferons, cancer immunotherapy, immune responses, resistance mechanisms, tumor microenvironment

## Abstract

The efficacy of several therapeutic strategies against cancer, including cytotoxic drugs, radiotherapy, targeted immunotherapies and oncolytic viruses, depend on intact type I interferon (IFN) signaling for the promotion of both direct (tumor cell inhibition) and indirect (anti-tumor immune responses) effects. Malfunctions of this pathway in tumor cells or in immune cells may be responsible for the lack of response or resistance. Although type I IFN signaling is required to trigger anti-tumor immunity, emerging evidence indicates that chronic activation of type I IFN pathway may be involved in mediating resistance to different cancer treatments. The plastic and dynamic features of type I IFN responses should be carefully considered to fully exploit the therapeutic potential of strategies targeting IFN signaling. Here, we review available evidence supporting the involvement of type I IFN signaling in mediating resistance to various cancer therapies and highlight the most promising modalities that are being tested to overcome resistance.

## Introduction

Cancers exhibit remarkable phenotypic and functional heterogeneity and various factors including genetic and epigenetic changes participate (1). The proposition is that different populations with higher or lower tumorigenic potential co-exist in tumors where stem cells occupy the pinnacle of the hierarchy (2). Cancer stem cells are now known to possess therapy resistant properties, and not only do they exist in the tumors prior to treatment but non-stem cells can also acquire stem cell properties post treatment conferring further resistance. However, this intrinsic plasticity is not the only mechanism of acquired resistance to therapy. The heterogeneous and constantly evolving composition of the tumor microenvironment also actively contributes to cancer progression including modulation of resistance to therapies.

From an immunological point of view, cancer development and progression depend on the cross talk between tumor cells and immune cells. Cancers evade anti-tumor immune responses through different mechanisms, which include the induction of local immune-suppression while an inflammatory state is simultaneously maintained. Cytokines are important in the cross-talk between tumor cells and immune cells. Interferons (IFNs) comprise a large family of cytokines that have been studied extensively in the context of virus infections but they are now also known as key drivers of inflammation within the tumor microenvironment (3). IFNs have critical immune-stimulatory effects on various immune cells including tumor-specific T lymphocytes (4). However, evidence emerged in the last years suggests that IFNs may also trigger immune suppressive mechanisms in cancer, highlighting an additional important mechanism exploited by cancer cells to promote malignant progression and resist therapies. Therefore, dissecting the role of IFNs and associated signaling pathways in the complex and dynamic interplay between the tumor and its surrounding microenvironment is critical to tailor therapeutic intervention.

IFNs play a key role in many biological processes, whether its immune responses against pathogens and cancers or cell differentiation and apoptosis (5). IFNs are of three different types: type I (α, β, ε, κ, and ω), which bind IFNα/β receptor 1 (IFNAR1), and IFNAR2 subunits, type II (γ) binding IFN-γ receptor 1 (IFNGR1), and type III (λ), which binds the IFN-λ receptor 1 and the IL10 receptor subunit β heterodimeric receptor. We herein focus on Type I IFNs, which are critical determinants of the efficacy of anti-tumor immunity. The immune-stimulatory properties of type I IFNs, including the stimulation of dendritic cell maturation, the enrichment in granzyme and perforin expression in cytotoxic T-lymphocytes and the enhancement of memory T-cell survival, make these cytokines essential in cancer immunosurveillance (6). Moreover, type I IFNs have direct inhibitory effects on tumor cells of various origin as they limit their proliferation and drive senescence and apoptosis. It is now clear that this inhibition is attained by a combination of cell cycle arrest and cell death. Similar effects are also seen on proliferating endothelial cells during tumor angiogenesis (7). However, under certain circumstances, Type I IFNs may also trigger opposite effects, thus resulting in evasive mechanisms and promotion of tumor progression (8).

It is well established that the efficacy of several therapeutic strategies against cancer, including cytotoxic drugs, radiotherapy, targeted immunotherapies and oncolytic viruses, depend on intact type I IFN signaling (6) for the promotion of both direct (tumor cell inhibition) and indirect (effective anti-tumor immune response) effects. Malfunctions of this pathway in the tumor microenvironment or in immune cells may be causative factors behind therapeutic resistance in cancer patients. On the other hand, type I IFNs may mediate immune-suppressive effects, as in case of infection, where chronic persistence of the pathogen triggers type I IFN-induced negative regulatory pathways (8, 9). Recent evidence indicates that similar negative effects may also occur in cancer-associated chronic inflammation, where chronically type I IFNs-activated signaling may be involved in mediating resistance to treatments (8, 9). Here, we review available evidence on the contribution of type I IFN signaling in resistance to various cancer therapies and highlight some of the modalities being tested in the lab and clinic to overcome resistance.

## Type I IFN signaling and its modulation

Type I IFN signaling and its modulation has recently been reviewed in detail elsewhere (10, 11). We herein highlight some of the components and regulators of this pathway that may affect the outcome of common forms of cancer therapies. The Type I IFN family includes a single isoform of IFN-β, multiple variants of IFN-α and other less studied variants, like IFN-ε, IFN-κ, and IFN-ω (12). While IFN-β is produced by most cells, IFN-α is primarily released by plasmacytoid dendritic cells (13). Type I IFNs are secreted by infected cells following the recognition of microbial products by pattern recognition receptors (PRRs), which include transmembrane Toll-like receptors (TLRs) recognizing damage associated molecular patterns (DAMPs) and pathogen associated molecular patterns (PAMPs). Other than TLRs, cytoplasmic sensors such as cyclic GMP-AMP synthase (cGAS), RIG-I like receptors, MDA-5, DDX41, and DAI can recognize viral and tumor nucleic acids (14). Once these sensors have been activated, they interact with adaptor proteins, such as TIR-domain-containing adaptor inducing IFN (TRIF), MyD88 adapter-like (Mal), mitochondrial anti-viral signaling protein (MAVS) or STING (15). TRIF and MyD88 recruit ubiquitin ligases that then activate kinases while STING directly recruits kinases like TANK-binding kinase 1 without the need of ubiquitin ligases. These kinases phosphorylate IFN regulatory factor 3 (IRF3), AP-1 and NF-κB triggering their translocation into the nucleus, where they bind to the regulatory domains of the IFN-β gene promoter (16). The production of most IFN-α types, on the other hand, requires constitutive expression of the IRF7 transcription factor instead of IRF3 (17).

Upon production, type I IFNs signal via a transmembrane receptor composed of IFNAR1 and IFNAR2 subunits. Canonically, upon binding to IFN, IFNAR phosphorylates and activates the receptor-associated Janus kinase 1 (JAK1) and tyrosine kinases 2 (Tyk2), which subsequently lead to the phosphorylation of signal transducer and activator of transduction 1 and 2 (STAT1 and STAT2) present in the cytosol. Upon activation, these proteins dimerize, get translocated to the nucleus and bind to IRF9 to form a STAT1-STAT2-IRF9 complex (ISGF3) (18). This complex then binds to IFN stimulated response elements (ISRE) in the promoter region of IFN-stimulated genes (ISG), leading to the activation of ISG transcription, most of which contribute to immune-stimulatory and anti-viral effects. Non-canonical pathways of type I IFN signaling can be mediated by STAT1 homodimers (19). STATs associated with other cytokines signaling, including STAT3, STAT4, STAT5A, and STAT5B can also mediate type I IFN signaling and expression of various ISGs (13).

ISGs are responsible for various immune-modulatory activities. PRRs, JAKs, and STATs are also ISGs and may re-inforce IFN signaling. Type I IFN-upregulated ISGs include genes required for the expression of mature MHC class I complex such as those encoding for β_2_ -microglobulin. Other ISGs, such as SECTM1, may act as co-stimulatory ligands for T cells after TCR activation. Several ISGs, like IFITM proteins, IFIT proteins, GBP1, IFI6, IFI27, IRF1, IRF9, ISG20, MX1 or MXA, OAS1, PKR, PML, and viperin, have direct anti-viral activity (20). A few of these ISGs, like MxA, have now been identified as tumor suppressors in cancer, whereas the role of other ISGs in the tumor microenvironment has not been characterized yet. A number of ISGs, such as CCL5, CXCL10, CCL3, and CCL9 function as chemo-attractants to lymphocytes and monocytes. There are also various ISGs endowed with pro-apoptotic effects, including TRAIL, Fas/FasL, XIAP-associated factor-1(XAF-1), OAS, ISG12 (IFI27), and death-activating protein kinases (DAP kinase), phospholipid scramblase (PLSCR1) and IRFs (21). PLSCR1 also encodes for a protein required to provide macrophages with a signal to engulf debris after tumor cell apoptosis and is also a negative regulator of autophagy (22). Other ISGs participate in negative regulation of IFN signaling as is the case of USP18, whose interaction with IFNAR2 results in decreased stability of the IFN-IFNAR binding (23). USP18 also participates in removing ISG15 from its substrates (ISGylation) (24), which is known to promote type I IFN production and secretion (25). Among other regulatory ISG proteins, SOCS1 and SOCS3 are known to negatively regulate both type I and type II IFN JAK-STAT signaling pathways (11).

**Figure 1:**
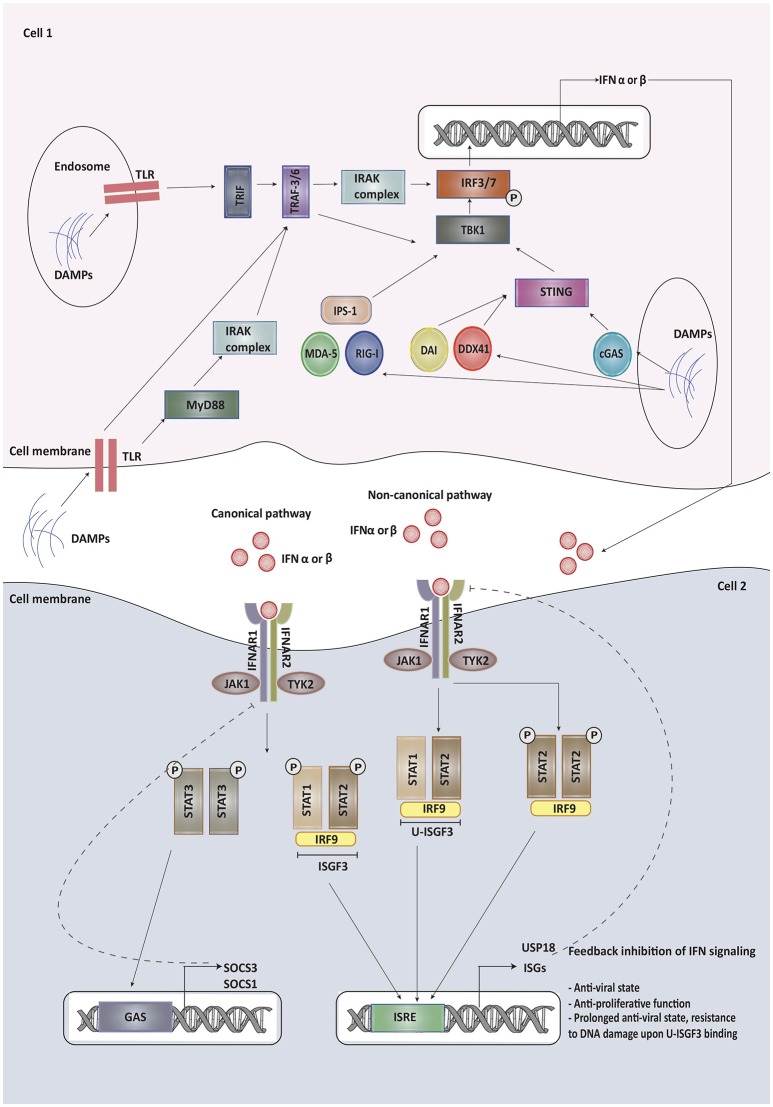
Type I IFNs are secreted by infected cells upon recognition of damage associated molecular patterns (DAMPs) and pathogen associated molecular patterns (PAMPs) by pattern recognition receptors (PRRs), which include transmembrane Toll-like receptors (TLRs), cyclic GMP-AMP synthase (cGAS), RIG-I like receptors, MDA-5, DDX41, and DAI. Once these sensors have been activated, they interact with adaptor proteins, such as TIR-domain-containing adaptor inducing IFN (TRIF), MyD88 adapter-like (Mal), mitochondrial anti-viral signaling protein (MAVS) or STING. These adapter proteins recruit kinases indirectly or directly, which then phosphorylate IFN regulatory factor 3/7 triggering its translocation into the nucleus, where it binds to the regulatory domains of the IFN-β/α gene promoter. Upon production, type I IFNs signal via a transmembrane receptor composed of IFNAR1 and IFNAR2 subunits. Canonically, upon binding to IFN, IFNAR phosphorylates and activates the receptor-associated Janus kinase 1 (JAK1) and tyrosine kinases 2 (Tyk2), which subsequently lead to the phosphorylation of signal transducer and activator of transduction 1 and 2 (STAT1 and STAT2) present in the cytosol. Upon activation, these proteins dimerize, get translocated to the nucleus and bind to IRF9 to form a STAT1-STAT2-IRF9 complex (ISGF3). This complex then binds to IFN stimulated response elements (ISRE) in the promoter region of IFN-stimulated genes (ISG), leading to the activation of ISG transcription, most of which contribute to immune-stimulatory and anti-viral effects. Some of the ISGs provide feedback inhibition of type I IFN signaling. Non-canonical pathways of type I IFN signaling can be mediated by STAT homodimers or unphosphorylated STAT1-STAT2 heterodimers leading to the formation of unphosphorylated ISGF3 (U-ISGF3). U-ISGF3 maintains the subset of ISGs whose production leads to DNA damage resistance. SOCS proteins produced on binding of phosphorylated STAT3 homodimers to GAS promoter are involved in providing negative regulation to type I IFN signaling.

A detailed overview of type I IFN signaling and its regulators is depicted in Figure 1.

## Common cancer therapies and type I IFN signaling

### Cytotoxic therapies

Radiotherapy (RT) has long been used for curative treatment for various forms of cancer. Besides its direct cytotoxic activity, indirect effects of RT on tumor cells via immune-mediated mechanisms system have been also reported. A study dated back to 1979 showed that the therapeutic efficacy of RT is determined by the host immune status (26). Some cytotoxic therapies may cause the release of tumor-associated nucleic acids and stress proteins by dying cells that may lead to the activation of TLRs in immune cells. Of particular therapeutic relevance is the recently emerged concept of immunogenic cell death (ICD) induced by several antineoplastic drugs and radiotherapy, which is characterized by the release of DAMPs that promote immune activation. Additionally, HMGB1 released during ICD may activate TLR4. As described above, these signals promote Type 1 IFN secretion.

The importance of type I IFNs in RT-mediated tumor suppression was first revealed in a study by Burnette et al. showing that type I IFN produced by myeloid cells in a mouse melanoma model was essential for tumor eradication following RT (27). Subsequently, these observations were confirmed in another pre-clinical model showing that radiation-induced type I IFNs increased CXCR3 levels, which assists in the recruitment of lymphocytes at tumor site, showing a role of type I IFN in radiation-induced ICD (28). Using an inducible expression system in tumor cells, this study also showed that exogenously administered IFN-α levels further enhanced therapeutic efficacy of RT. Experiments carried out in STING knockout mice and conditional knockouts of IFNAR1, demonstrated that activation of cytosolic DNA sensing pathways in DCs was required for the induction of type I IFN responses in DCs. The same study also showed that type I IFN signaling was required for the DCs to cause the activation of CD8^+^ T-cells to achieve a therapeutic response. STING and cGAS, but not MYD88 or TRIF, were shown to be required for the ability of radiation to induce type I IFN responses.

Over the last decade, it has become clear that RT can enhance innate and adaptive immune responses to tumors by triggering ICD and that localized radiation may trigger systemic antitumor effects, the so-called “abscopal effect” (ab scopus i.e., away from the target). Although the occurrence of the abscopal effect is relatively rare in the clinic, with the progressive development and use of novel immunotherapy strategies incorporating RT, the abscopal effect is becoming increasingly relevant in the treatment of a variety of human tumors (29).

As in the case of RT, the anti-cancer benefits of chemotherapy were initially thought to be solely the effects of direct cytotoxicity or cell cycle arrest. However, research over the last 10 years has convincingly shown that chemotherapy can also lead to ICD, which may actively contribute to the induction of therapeutically relevant anti-tumor immune responses. Casares et al. showed that injection in mice of cancer cells treated with doxorubicin *in vitro* prevented the *in vivo* growth of the same tumor cells in challenged mice, consistently with the induction of an effective anti-tumor immune response (30). Several other drugs used in the clinic as monotherapies or in combination, such as anthracyclines (doxorubicin, epirubicin, mitoxantrone, bleomycin) and oxaliplatin have also been shown to induce ICD, while etoposide, mitomycin C, and cisplatin do not (31). Interestingly, the immune-mediated effects induced by some drugs correlate with the chemotherapeutics that are more effective in the clinic than the others (32). Of note, ICD induction by anthracyclines is strictly dependent on their ability to promote the activation of IFN-dependent gene expression programs in tumor cells that promote the generation of effective anti-tumor immune responses (33).

Indeed, release of Type 1 IFNs by tumor and immune cells induced by various chemotherapy and RT regimes can stimulates an adaptive immune response against dead tumor cell-associated antigens via autocrine and paracrine activation of the IFN signaling pathway. Sistigu et al. showed the critical role of type I IFN response activation in tumor cells by ICD inducers and demonstrated that anthracyclines stimulate TLR3 in cancer cells prompting a type I IFN signaling pathway (34). Type I IFNs were shown to be produced by cancer cells 1–4 days after chemotherapy, when the accumulation of dying cells starts. Doxorubicin was found to increase transcript levels of several ISGs, including Rsad2, Mx2, OAS2, IRF7, IFIT2, and intriguingly, CD274, the PD-L1-encoding gene. IFN-α and -β, when exogenously supplied, also enhanced the therapeutic activity of the non-ICD inducer cisplatin (34) showing that type I IFNs and activation of IFN signaling pathway may lead to ICD-like effects. A type I IFN-related signature was shown to predict clinical responses to anthracycline-based chemotherapy in several independent cohorts of patients with breast carcinoma characterized by poor prognosis. This study also outlined the potential relevance of the IFN-stimulated GTP-binding protein MX1 in mediating the efficacy of anthracycline-based chemotherapy. In fact, MX1 was upregulated by anthracyclines and its high expression levels were associated with better overall survival in breast cancer patients who received anthracycline-containing chemotherapeutic regimens (34). These observations indicate that “viral mimicry” response that features type I IFN signaling activation is a prerequisite for the success of immunogenic chemotherapy, and potentially also of RT.

### IFN-only therapies

Considering the pro-apoptotic, anti-angiogenic, and immunomodulatory actions of type I IFNs, they were expected to be the ultimate therapy against malignancies and infectious diseases. Indeed, type I IFN therapies initially proved successful in comparison to conventional chemotherapies for the treatment of cancers like leukemias, lymphomas, and myelomas. In chronic myeloid leukemias (CML), complete cytogenetic response was achieved in 20–30% of the cases and increased survival was observed (35). However, systemic toxicity and poor tolerability strongly limited the clinical use of these cytokines. Interestingly, IFNs have made a comeback for CML in clinical trials. A recent study investigated CML patients on IFN-α therapy and found prolonged complete molecular response, a sought-after goal in CML therapy, and very low risk of relapse in comparison to patients treated with targeted therapy (Imatinib) (36). The authors attributed these observations to IFN-induced activation of cell-mediated immunity to leukemic stem cells, a feature not seen with Imatinib. Other clinical trials have indicated that the combination of IFN-α with Imatinib is more effective for these patients in comparison to Imatinib alone (37–39).

Systemic administration of type I IFN in breast cancer mouse models has shown decrease in tumor progression and metastasis to the bone and prolonged metastasis free survival via NK-cell anti-tumor function (40, 41). However, in the clinic, treatments with type I IFN for breast cancer, melanoma and renal carcinoma have shown moderate success in terms of clinical responses and tolerability. Moreover, for breast cancer, a combination of IFN-α and IFN-β has been tested in many randomized trials owing to the demonstration that these drugs upregulate estrogen receptor (ER) in tumor cells (42). The possible ER up-regulation in ER-negative patients were thought to be able to convert them into responders to targeted therapy, but the results have been varied. Interestingly, the use of IFN-β with tamoxifen and retinoic acid showed better response rates in comparison to IFN-α in the same combination, suggesting that IFN-β might be a better anti-cancer agent than IFN-α in some clinical settings for breast cancer patients (43).

The aforementioned side effects associated with systemic administration of IFN include nausea, fatigue, fever dizziness, which can be managed with prophylactic acetaminophen (44), to more severe neuropsychiatric symptoms like depression, which are less manageable even with anti-depressants (45), making IFN a less favorable choice for therapy. Other factors that limit the efficacy of systemic IFN therapy include the limited bioavailability due to short systemic half-life (46). Therefore, overall response to type I IFN-therapy across various cancer types has been subpar due to the toxicity associated with systemic administration and the limited efficacy at the maximal tolerated doses, thus calling for less toxic and more effective delivery strategies.

Pegylation of IFN has been shown to efficiently resolve the half-life and bioavailability issues (47) by providing longer half-life and a persistent steady state of drug activity. Indeed, pegylated type I IFNs showed therapeutic efficacy in different preclinical models of cancer (48, 49). More recently, pegylated IFN-β was shown to significantly inhibit the vascular permeability of the peritoneal membrane in animal models of ovarian cancer and gastric cancer cell xenograft mice (50). In the clinical setting, however, unlike what observed in patients with chronic viral infections, the use of pegylated type I IFNs was associated with limited benefit. Adjuvant therapy with pegylated IFN-α was investigated in surgically resected stage III melanoma; at the mature median follow-up of 7.6 years, there was a significant but modest improvement in relapse-free survival but there were no significant benefits observed in overall survival or distant metastasis-free survival. Subgroup analysis suggested that the benefit of adjuvant pegylated IFN-α may be confined to the group of patients with microscopic nodal metastasis, and among this group, patients with an ulcerated primary may have benefited the most. With regards to tolerability, 37% of patients discontinued adjuvant therapy because of limiting toxicities (51). More recently, a randomized phase III trial including patients with resected cutaneous melanoma stage IIA-IIIB showed that pegylated IFN-α did not improve the outcome over IFN Notably, a higher percentage of patients under pegylated IFN-α discontinued treatment due to toxicity (52).

As an alternative way to reduce systemic toxicity and deliver IFN-α in a tumor-targeted manner, we developed a gene and cell based therapy where we engineered hematopoietic progenitors so that the expression of an IFNα transgene was restricted to their monocytic progeny, including tumor-infiltrating macrophages. Activation of innate and adaptive immune cells was seen in mice chimeric for these IFN-α-expressing macrophages and disease progression was inhibited in mouse and humanized mouse models of breast cancer with no evident signs of toxicity (53–55).

### Immune checkpoint inhibitor therapies

While RT, chemotherapies and surgery have been traditional choices for treatment of cancer, immunotherapies have revolutionized cancer therapeutics over the last decade. The benchmark in immunotherapies has been set by the immune-checkpoint inhibitors (ICI) cytotoxic T-lymphocyte-associated antigen-4 (CTLA-4) and programmed death-1 (PD-1) blocking antibodies. Chimeric antigen receptor (CAR)-T cell therapy is a more recent addition to the list of immunotherapies, and it is making headlines due to its novelty and efficacy mainly in the treatment of hematological malignancies.

Inhibitory immune checkpoint molecules are important regulators of the immune system that specifically controls the levels of T cell activation to avoid excessive inflammation and ensure self-tolerance. The immune checkpoint CTLA-4 is expressed exclusively on T-cells where it modulates early stages of T-cell activation by counteracting the activity of the T-cell costimulatory receptor CD28. CTLA-4 and CD28 share identical ligands CD80 and CD86 expressed by APCs. Full activation of T-cells requires binding of CD28 to CD80 and CD86. Upregulation of CTLA-4 dampens T-cell activation through sequestration of CD80 and CD86 from CD28 engagement. While CTLA-4 was the first immune checkpoint to have been clinically exploited, the PD-1/PD-L1 axis has more recently garnered a higher amount of interest. Similar to CTLA-4 signaling, PD-1 regulates T-cell activation by binding to its ligands, called programmed death ligand-1 and−2 (PD-L1 and PD-L2). High and persistent PD-1 expression is characteristic of exhausted T-cells that have undergone high levels of stimulation or have experienced suboptimal CD4 T-cell help (56).

In cancers as well as in persistent infections, T-cells chronically exposed to antigen upregulates inhibitory checkpoints molecules which weaken their effector functions thus allowing the disease to escape anti-tumor immunity and ultimately progress. These exhausted T cells are unable to perform their effector functions against persisting tumors and pathogens optimally. By blocking the interaction of CTLA-4 and PD-1 with their ligands expressed by tumor cells or by antigen presenting cells (APCs), the effector functions of exhausted T cells can be at least partially revived to provide protective immunity. This blockade is provided by monoclonal antibodies targeting CTLA-4 and PD-1/PD-L1, the generalized term for which is “immune checkpoint inhibitor therapy.”

Monocloncal antibodies targeting CTLA-4 and PD-1/PD-L1 have been approved for use in the clinic for non-small cell lung cancer, renal cell carcinoma, melanoma, Merkel's cell carcinoma, Hodgkin lymphoma and various other malignancies (57). Despite proven utility as a therapy in over 15 cancer types, clinical efficacy of PD-1/PDL-1 monotherapy rarely exceeds 40% and a large number of partial and non-responders are observed (58). Similarly, FDA-approved anti-CTLA4 ipilimumab results in significant survival benefit for only 20% of the metastatic melanoma patients (59). This may be due to primary resistance developed because of tumor-intrinsic genetic and epigenetic factors. However, the responders can also acquire resistance to the therapy after an initial response.

The efficacy of ICI depends on the augmentation of immune responses by enhancing either the activity or number of CTLs which can target tumor cells. Type I IFNs are critically involved in the activation of innate and adaptive immunity required to promote anti-tumor immune responses by both autocrine and paracrine mechanisms (60). IFNs promote survival, immunoglobulin class switching in B cells, CD8^+^ T-cell proliferation and cytotoxicity and activation of dendritic cells (DCs), which have a crucial role in the initiation of adaptive immune responses. Furthermore, IFNs increase natural killer (NK) cell cytotoxicity by enhancing NK cell survival, modulating the surface expression of activating and inhibitory receptors, and NK cell expansion by inducing IL-15 production. Finally, type I IFN-mediated activation of the STING pathway post cytosolic DNA sensing is one of the key players in sustaining a T-cell inflamed-tumor phenotype (61). Activation of this pathway contributes to the activation of Batf3^+^ dendritic cells, central to antigen presentation and hence to T-cell effector functions. Given the pleiotropic activity of IFNs in controlling maturation survival and activation of most immune cells, they are expected to play an important role in mediating therapeutic responses to ICI. At the same time, as described below, counteracting regulatory mechanisms within the immune system and mediated by chronic exposure to type I IFNs, such as upregulation of inhibitory checkpoint molecules, can negatively affect the anti-tumoral efficacy of ICI.

### CAR-T cell therapies

CAR-T-cell therapies are characterized by a more targeted approach than checkpoint inhibitors. They rely on re-directing T-cell function to a tumor-specific antigen through the expression of a chimeric antigen receptor (CAR). CARs consist of a variable fragment of an immunoglobulin for antigen recognition, linked to T-cell activation (CD3ζ) and co-stimulation (CD28, CD137, and CD134) intracellular signaling domains (62). T-cells are derived from the patients, modified in the laboratory to express antigen specific CARs and infused back into the patients. Upon antigen recognition, the T cells become activated and eventually lyse the target tumor cells. CAR-T cells have shown significant promise with a dramatically high remission rate in various hematological malignancies, particularly B-cell acute lymphoblastic leukemia (63). The importance of type I IFN pathway in modulating CAR-T-cell efficacy was demonstrated by the work of Katlinski et al. They have tested CAR-T cells against fibrinogen activated protein (FAP) generated from lymphocytes of mice with normal and downregulated IFNAR, and showed that IFNAR1 downregulation on CTLs compromised their viability and hence their function in the tumor microenvironment (64). CAR-T cells from mice with downregulated IFNAR1 were also less effective against colorectal adenocarcinomas in mice and this effect was dependent on p38α, a kinase involved in ligand independent downregulation of IFNAR1 (64). These findings warrant further exploration of type I signaling in solid tumors where CAR-T cell therapies are still poorly effective (65).

### Oncolytic virus therapy

Oncolytic viruses (OV) represent a new class of therapeutic agents that stimulate anti-tumor responses by selective tumor cell killing and induction of systemic anti-tumor immunity. These viruses can selectively target and kill cancer cells without causing damage to the surrounding normal tissue. There are two main types of OV. The first type replicates preferentially in cancer cells but not in normal human cells due to increased sensitivity to anti-viral pathways or their dependence on oncogenic pathways and includes poxviruses and paramyxovirus. The other OV type is genetically engineered with mutations preventing replication in normal but not in cancer cells and includes adenoviruses (Ad), herpes simplex virus (HSV), and vesicular stomatitis virus (VSV).

Several studies have shown the efficacy of OVs in the treatment of various cancers. To date, two oncolytic viruses have been approved for use in clinic, Oncorine, a *E1B*-deleted adenovirus approved for head and neck cancers in China (66), and Talimogene Laherparepvec (T-Vec), an HSV-based virus, approved for melanoma in Europe, Australia and USA (67). However, treatment with these viruses can result in side effects and patients can develop resistance (68). Therefore, new generation of OVs are now being tested in preclinical studies and Phase II or III clinical trials (69), VSV being one of the most explored types. OV therapy is particularly interesting regarding the possible involvement of type I IFN because the success of this treatment strongly depends on the presence of a dysfunctional IFN signaling often found in cancer cells, as these viruses are susceptible to IFN-mediated antiviral activity. This constitutes a distinctive feature of OV, not present in all the other therapeutic approaches mentioned above. A “proof of concept” was provided by various studies that have shown the association of oncolytic properties of viruses with defective IFN signaling in cancer cells. In particular, Hummel et al. showed that HSV-1 could destroy murine breast carcinomas, which were defective in producing and directly responding to IFN (70). Other studies have reported an increase in sensitivity of cancer cells to VSV-induced cancer cell death upon knock-down or blockade of IFN pathway components, including IFNAR (71), IRF5, and IRF7 (72) in the tumor cells.

## Mechanisms of resistance to type I IFNs

With the exclusion of OV therapies that benefit from a dysfunctional IFN signaling, other therapies requiring active IFN signaling to elicit an anti-tumoral activity can develop two main forms of resistance: (i) silencing of the IFN signaling pathway or (ii) counter-regulatory mechanisms blocking the effects of an active IFN signaling pathway.

Silencing of type I IFN signaling will inhibit the direct effects of IFN on tumor or immune cells, such as inhibition of cell proliferation. However, it will also affect the IFN-induced cross talk between tumor cells and the immune system thus indirectly impairing an effective anti-tumor immune response. Counter-regulatory mechanisms are mostly seen within the immune system and derive from normal physiological mechanisms that modulate and control excessive inflammatory and immune responses.

Since IFNs are central to the efficacy of various cancer therapies, general mechanisms of resistance to Type I IFNs could identify and explain several modalities of resistance across other therapies such as cytotoxic therapies or immunotherapies.

### Resistance due to loss of IFN signaling

#### Resistance to IFN-only therapies

##### Down-regulation of IFNAR1

Down-regulation of IFNAR1 in immune and tumor cells as a mechanism of resistance to IFN in cancers has, perhaps deservedly, garnered most interest in this context as cell surface IFNAR1 levels are key for type I IFN anti-proliferative effects (73). IFNAR1 degradation is brought about by ubiquitination, facilitated by E3 ubiquitin ligases, which bind to phosphorylated serine in IFNAR1. The phosphorylation of these serine residues has been shown to be triggered by vascular endothelial growth factor (74), oxygen deficit (75, 76) and the pro-inflammatory cytokines TNFα, IL-1 ad IL-6 (76), factors which are all present within the TME. Serine phosphorylation of IFNR1 eventually leading to its downregulation is also stimulated by virus-induced unfolded proteins (77), a prominent feature of TME in many cancers [Reviewed by Vanacker et al. (78)]. Recently, Katlinski et al. observed a complete or partial loss of IFNAR1 in all cell types of human colorectal adenocarcinomas compared to normal human colon cell types (64). Using mice deficient in IFNAR1 ubiquitination and degradation, this study showed that the downregulation of IFNAR1 stimulated tumorigenesis by altering the expression of IFN-induced genes including *Irf7, Ifit2, Mx2, Usp18*. Downregulation of IFNAR1 was also seen in T-cells, which showed weakened survival in the TME through suppression of the IL-2 pathway. Similar observations have been reported for melanomas (79). IFNAR1 downregulation not only causes resistance to IFN monotherapies, but has also been associated with resistance to chemotherapy (80) and immune-checkpoint inhibitor therapies (64) (see below).

##### Upregulation of SOCS proteins

SOCS proteins have also been implicated in silencing of type I IFN signaling. Cancer cells can upregulate the expression of SOCS1 and SOCS3 proteins which leads to a decline in IFN-induced STAT1 phosphorylation (81). Indeed, experimental SOCS1 and SOCS3 over-expression resulted in type I IFN unresponsiveness, while their inhibition re-invigorated responsiveness and expression of ISGs, IFIT2 and ISG-15. Consistently, silencing of SOCS1 increases the sensitivity of neuroendocrine tumor cells to type I IFNs (82). SOCS1 mRNA was also associated with poor cytogenetic responses to IFN-α and shorter median progression-free survival in CML patients (83). Similarly, silencing of SOCS3 increases the susceptibility of renal cell carcinomas to IFN (81). However, both proteins were shown to also increase the sensitivity to IFN in certain cancer types. What determines such an opposite effect is still unknown (84).

##### Jak-STAT signaling modulation

Variability in the role of Jak-STAT signaling components in resistance to type I IFN has been seen across various studies. Different components seem to play a role in different cellular backgrounds and in different tumor types. Epigenetic silencing of JAK1 conferred IFN-α unresponsiveness in prostate adenocarcinoma cell lines (85). Loss of STAT2 and defective ISGF3 mediated gene activation were linked to resistance to IFN-α induced apoptosis (86). Subsequently, defects in ISGF3 caused resistance to IFN-α in HCC was shown to be due to the absence of the p48-ISGFγ protein (87). A lack of STAT1 expression has been shown in CML patients resistant to IFN-α (88). Further, STAT5 overexpression has been reported in IFN-α resistant melanoma cells and advanced melanoma lesions (89). A study showed the association of the lack of Stat1, Tyk2, and Jak1 expression and defective Jak-Stat activation with resistance to IFN-α in renal cell carcinoma cells, while IFNAR1 and SOCS3 proteins were not involved (90).

##### Silencing of IRF genes

The success of type I IFN therapies strongly depend on the immunomodulatory properties of IFN, which are mainly regulated by IRF7. Suppression of IRF7-regulated genes was shown to be crucial for the induction of bone metastasis in breast cancer, while restoration of IRF7 in tumor cells or administration of IFN, reduced metastasis in mice in a NK and CD8^+^ T-cell dependent manner (91). Similarly, overexpression of IRF7 reduces bone metastasis in mouse models of prostate cancer (92). Loss of IRF5 has also been shown to correlate with disease stage and metastasis in cancers and may constitute another mechanism underlying resistance of advanced tumors to IFN-therapies (93).

##### Overexpression of miRNAs

Other factors involved in resistance to type I IFNs are miRNAs. Tomimaru et al. showed that miRNA-21, which is overexpressed in hepatocellular carcinoma (HCC), can induce resistance to IFN-α. miRNA-21 expression was also higher in non-responders to a combination of IFN-α and chemotherapy (94). Following these results, Tomokuni et al. carried out a comprehensive expression profiling of miRNAs in HCC cells and their IFN-α resistant clones, and found that miR-146a could also suppress the sensitivity of these cells to IFN-α (95). Interestingly, these miRNA have also been shown to induce resistance to chemotherapy (96).

#### Resistance to ICI due to loss of IFN signaling

Despite the transformative potential of immune checkpoint-based immunotherapies, upfront clinical benefits in approved indications are not seen in all patients or even all cancer types. Additionally, resistance to these drugs still constitutes a relevant factor limiting the efficacy of ICIs. Recent studies have indicated impairment of type I IFN signaling as one of the mechanisms behind acquired resistance to ICI therapies. Downregulation of IFN signaling would prove beneficial to the tumor in the presence of ICIs as blockade of inhibitory checkpoint pathways prevents exhaustion of T-cells while reduction of IFN signaling would reduce antigen presentation and further activation of T-cells.

This elegant mechanism of acquired resistance was recently revealed in patients treated with anti-PD-1 therapy. Zaretsky et al. performed molecular analyses on tumor tissues from four melanoma patients who showed an initial objective response to the PD-1 inhibitor pembrolizumab administered for 6 months followed by disease relapse (97). Out of these, two patients showed loss of function mutations in genes encoding JAK1 and JAK2 in the relapsed tumors, which were not present before treatment. When the functional effects of these mutations were tested, the authors found a total loss of functional response to IFN-γ but not IFN-α and β in the presence of JAK2 mutations while resistance to all three interferons was seen in the presence of JAK1 mutations (97).

A case of primary resistance to PD-1 and CTLA-4 blockade due to defects in IFN-γ signaling has also been described (98). Additionally, the loss of IFN-γ pathway genes IFNGR1, IFNGR2, JAK2, IRF1, IFIT1, IFIT3, MTAP, miR31 and amplification of the suppressor genes SOCS1 and PIS4 have been shown in melanoma patients non-responsive to anti-CTLA4 therapy. Interestingly, deletions in IFNA and IFNB genes are also seen in these patients, but the functional significance of this has not been tested (59). Missense mutations in IFNAR2 along with mutations in IFN-γ signaling pathway genes were also found in lung tumors that had acquired resistance to PD-1 blockade (99). The loss and mutation of genes overlapping between type I and type II IFN pathways and loss of IFNA and IFNB might suggest a role for Type I IFNs in resistance to ICIs and calls for further exploration.

#### IFN resistance and OVs

Unlike other therapies, resistance to IFN helps the therapeutic efficacy of OVs. A role of type I IFNs in resistance to OVs was highlighted by a study on HCC cells, where impairment of type I IFN signaling resulting from a deregulated IRF3 pathway conferred susceptibility to VSV infection (100). In another study, VSVs were tested on a panel of aggressive pancreatic ductal adenocarcinoma cell lines and 5 cell lines that showed resistance to VSV were not only sensitive to IFN-α treatment but also capable of secreting IFN-β (101). Subsequently, it was found that there was no difference in IFNAR expression between resistant and sensitive cells, but a great variability in the expression of ISGs, MxA, and OAS, with resistant cells showing high expression levels of these genes (102). Other studies have also shown a role for these ISGs in mediating resistance to OVs (103, 104). A recent study has reported an increase in tumor cell sensitivity to VSV induced by downregulation of the MX1 gene (105). The PML gene has also been implicated in resistance to OVs (106), whereas the role of other ISGs largely remains unexplored.

### Resistance due to chronic exposure to IFN

#### Resistance to ICI due to chronic exposure to IFN

Benci et al. showed that, upon prolonged IFN-γ exposure (but not type I IFN), B16 melanoma cells adopt a state of STAT1-dependent resistance to ICI associated with the expression of the ISGs IFIT1 and MX1 (107). The authors showed PD-L1 dependent and independent resistance mechanisms in patients and mice treated with RT and CTLA-4. IFNAR knock-out studies demonstrated that type I IFN signaling is required to sustain resistance to PD-L1 blockade, but not for its induction, but the exact underlying mechanism remains unclear (107). These reports seem contradictory to the observations reported by Zaretsky et al (97) mentioned above but highlight the importance of timing in assessing functional responses. This study shows that ablation of IFN signaling on B16 cells enhances resistance exclusively upon delayed scheduling of dual CTLA-4 and PD-1 therapy. The study also showed that a delay in administration of JAK inhibitors or IFN receptor ablation on tumor cells promoted the induction of complete responses to ICI in resistant melanoma and breast cancer, again demonstrating the importance of scheduling in combination therapies (97).

Notably, type 1 IFNs have been shown to up-regulate the expression of the immune checkpoint molecule PD-L1 in tumor cells. Based on this premise, a recent study investigated whether PD-L1 could engage in abrogating IFN-mediated toxicity. PD-L1 reduced, but did not completely abrogate, IFN cytotoxicity and was found to protect cells by inhibitory crosstalk with type I IFN signaling pathway, particularly by inhibiting STAT3 upregulation (108). Expression of PDL-1 in tumor cells has also been associated with radio-resistance (109). Katlinski et al also showed that while downregulation of IFNAR1 in the cytotoxic lymphocytes in the TME can lead to an immune-suppressive environment, a stable IFNAR1 also caused an increased expression of PD-L1 on tumor cells (64). Altogether, these findings suggest that the continuous exposure of type I IFNs may lead to PD-L1 expression by tumor cells, which then may promote immune resistance through interaction with PD-1^+^ immune effectors. This hypothesis, however, remains untested. Given that the interactions between IFNs and the PD1/PDL-1 axis have been brought to the forefront in the last few years (110), the involvement of IFN in PD1/PDL-1-mediated restraint of immune cells and hence in the resistance to checkpoint inhibitors remains likely.

#### IFN resistance and RT

Khodarev et al. reported isolation of radio-resistant squamous cell carcinomas (SCC) by multiple exposures to RT of a radio-sensitive parental tumor (111). Upon comparison of gene expression profiles between the sensitive and resistant tumors, 25 genes belonging to the IFN-inducible pathway were differentially expressed. Notably, STAT1 was the most highly expressed gene in resistant tumors and sensitive cells transfected with STAT1 developed radio-resistance. Although STAT1 activation is required to trigger anti-tumor immune responses, and therefore STAT1 deficiency may prevent the induction of anti-tumor immunity, persistent STAT1 activation may be associated with therapeutic resistance, as in the case of RT. Consistent with this possibility, a study carried out on resistant SCCs concluded that STAT1 is overexpressed in tumors adapted to continuous exposure to IFN, leading to the selection of tumor clones resistant to IFN-mediated cytotoxicity and RT effects (112). However, the mechanisms behind these observations were not explored. Again in keeping with these findings, breast, prostate and glioma cancer cells were shown to overexpress multiple IFN-related genes, including STAT1, when treated with multiple fractionated doses as compared to single dose of RT (113).

Following these reports, an IFN-related DNA damage resistance signature (IRDS) composed of 36 genes was found. The IRDS signature genes included the top 25% of genes that correlated with resistance in 34 NCI60 cell lines treated with radiation, indicating an association between IFN response genes and resistance to RT. It was also shown that patients with IRDS^+^ breast cancer exhibited recurrence of disease following mastectomy and adjuvant RT (114). The expression signature composed of 8 IRDS genes, STAT1, IFI44, IFIT3, OAS1, IFIT1, ISG15, MX1, and USP18, was also shown to predict poor outcomes in glioblastomas post RT (115). A direct role for of IFN-β was demonstrated in up-regulating the expression of these IRDS genes via un-phosphorylated STAT1 and IRF9 to cause resistance to DNA damage and RT (116).

#### IFN resistance and chemotherapy

Similarly to what observed for RT, chronic inflammation and prolonged type I IFN stimulation may also lead to the development of resistance to chemotherapy, as demonstrated for chronic viral infections (8). Indeed, the IRDS gene signature has been found to confer resistance to both chemotherapy and RT (114). Additional screening studies have shown the upregulation of STAT1 and some of the ISGs included in the IRDS signature are also upregulated in doxorubicin resistant cells (117). This dichotomy in the role of type I IFN signaling in resistance to these treatments may be due to the activation of signaling downstream of type I IFNs, driven by un-phosphorylated STAT1 and U-ISGF3 activated upon prolonged exposure, as genes upregulated by un-phosphorylated STAT1 (and not by phosphorylated STAT1) overlap with the IRDS (19). These findings, however, need further and direct investigation *in vivo* in pre-clinical models and in patient samples.

A recent study found a strong correlation between the genes belonging to the IRDS signature and genes upregulated in breast cancer cells after long term stimulation of CD95 (118), an inducer of stemness (119). Acquisition of stemness features is a widely accepted mechanism by which cancer cells become less sensitive to RT (120) and chemotherapy (121, 122). This study showed that type I IFNs (but not type II IFNs) were required for CD95-induced stemness and did so through the phosphorylation and activation of STAT1 and upregulation of the STAT1 targets PLSCR1, USP18, and HERC8. Blocking IFNAR1 and IFNAR2 in CD95 pre-treated luminal breast cancer cell lines resulted in inhibition of the CD95-induced phosphorylation of STAT1 and induction of the stemness marker SOX2 (118). This points toward a potential mechanism by which IFN signaling may induce resistance to RT. Another study showed that the growth of therapy resistant cancer stem cells was promoted due to STAT1 dependent antiviral signaling activated by exosomal transfer of RNA between stromal and basal breast cancer cells, which also correlated with IRDS expression (123). These observations, however, may differ among cancer types or subtypes as IFN-β signaling has recently been shown to repress cancer stemness in the triple negative breast cancer subtype (124). Further studies are therefore required to understand how type I IFN may induce opposite effects in this setting (124).

A role for the STING cytosolic pathway in promoting IFN-induced resistance has recently been demonstrated in breast cancer regrowth after treatment with genotoxic chemotherapeutic agents such as mafosfamide (125). STING pathway is typically activated in immune cells in response to infections, and this study showed that the activation of this pathway in breast cancer cell lines exposed to genotoxic stress was potentiated by chemotherapy. These findings confirmed that type I IFN pathway plays an important role in causing the up-regulation of ISG expression in cancer cells in response to chemotherapy and demonstrate that the STING pathway also contributes to type I IFN production mediated by STAT1 activation (125). Following a short-term exposure to chemotherapy, tumor cells exhibit slow-cycling, dormant and chemo-resistant populations. It has been shown that 20 days after treatment, these cell populations formed growing colonies following cell-cycle resumption. Silencing of STING after mafosfamide treatment of breast cancer cells delayed the appearance of growing colonies of surviving cells, showing that the STING/IFN/STAT1 pathway acts as a cellular mechanism of cancer cell survival and re-growth after the genotoxic stress of chemotherapy (125). Interestingly, this study identified one of the ISGs which was not included in the IRDS signature, PARP12, as a downstream contributor to STING mediated cancer regrowth and resistance. This protein is known to have roles in antiviral responses, however, the mechanisms underlying its effects on tumor survival are not known.

Taken together, available data show that activation of type I IFN signaling is essential for the therapeutic efficacy of checkpoint inhibitor and cytotoxic therapies, but prolonged activation of this signaling and availability at low levels can also lead to resistance to these therapies.

## Targeting type i IFN signaling pathway as a promising strategy to overcome resistance to cancer treatments

Current cancer therapies may fail to suppress tumor recurrence and metastasis due to the intrinsic plasticity of the tumor microenvironment that constantly evolves and adapts to escape the selective pressure of anti-cancer therapies. Understanding which evasive mechanisms are induced by different treatments is fundamental for the rational design of new combination treatments. Acquired resistance to IFNs represent one of the evasive mechanisms to several therapies, all requiring active IFNs pathway for optimal anti-tumor activity. Dysfunctional IFN signaling, not only impairs the direct effects of IFNs on tumor cells, but it may also interfere with their cross talk with the immune cells thus preventing IFN-mediated activation of an anti-tumor immune response.

Schematically, the strategies to overcome resistance in the context of type I IFN signaling can be divided into two categories: (1) Approaches to induce type I IFN signaling and (2) Approaches that block type I IFN signaling. The first category has been the subject of many clinical trials, whereas the second approach is based on relatively new findings and is yet to be explored in the clinic.

One of the approaches among those aiming at inducing/enhancing IFN signaling is to combine conventional therapies with IFN-only therapies. Direct exposure of the immune cells to IFN may bypass the tumor cells and directly activate the immune system. However, as discussed above, prolonged exposure to IFN might be harmful and cause further resistance to therapies. Although monitoring the timing of exogenous type I IFNs administration alongside other therapies has not been explored in the clinic yet, compounds targeting type I IFN signaling pathway in combination with other therapies have emerged, and proved to be an effective treatment strategy. Using agonists for any singular component to promote IFN secretion or the use of antagonists for molecules like STAT1 and STAT3 to overcome their effects of chronic IFN signaling could both prove beneficial in this setting.

STING agonists caught researchers' attention: Flavone acetic acid, 5,6-dimethyllxanthenone-4-acetic acid (DMXAA) and cyclic dinucleotides have all been tested to target STING *in vivo* and have shown promising results (126). A cyclic dinucleotide ADUS100 showed significant anti-tumor activity in the triple negative breast cancer 4T1 model (127). Another study showed ADUS100 also delayed tumor growth in HER2^+^ breast cancer. Moreover, a synergistic effect was seen when ADUS100 was combined with an anti-PD-1 antibody and an OX-40 agonist antibody where tumor clearance was seen in 40% of the mice compared to only 10% of the mice with ADUS100 treatment (128). STING agonists also showed increased tumor regression when combined with anti-PD1 antibody in a pre-clinical squamous cell carcinoma model (129). Based on the success in pre-clinical models, multiple clinical trials are ongoing to test STING agonist monotherapy or in combination with anti-PD1 antibodies (NCT03010176, NCT03172936). Similarly, other PRRs whose activation can result in type I IFN responses are also being targeted in several clinical trials (NCT03065023, NCT02828098).

Inhibitors of different JAK-STAT proteins have been of interest for a long time. Among STAT3 inhibitors, STATTIC was observed to sensitize human colon cancer cells to chemotherapy *in vitro* and *in vivo* (130). STX-0119, an inhibitor of STAT3 dimerization, was also shown to suppress the growth of lymphomas in mice (131). Additionally, the STAT3 inhibitor OPB-31121 displayed tumor suppression in pre-clinical models of gastric cancer (131) and mouse models of primary human leukemia. This inhibitor showed a high level of safety and tolerance in a clinical trial for patients with advanced solid tumors but has not been approved for clinical use (132). Meanwhile, STAT3 antisense nucleotides continue to be tested in combination with other therapies. AZ9150 has been shown to increase chemo-sensitivity and decrease tumorigenicity in other tumors *in vivo* (131). This inhibitor is now being tested in combination with durvalumab, an anti-PD1-PDL1 interaction blocking antibody, with and without chemotherapy in lung cancer patients (NCT03421353).

Pravastatin is a STAT1 inhibitor tested in various clinical trials that modulates type II IFN responses while its effects on type I IFNs remain undefined. Fludarabine, another STAT1 inhibitor, is now being tested with pegylated liposomal doxorubicin in patients with refractory ovarian cancer (NCT03335241). JAK1 and JAK2 inhibitor, ruxolitinib, was initially developed to target the inherent activation of JAK-STAT signaling pathway in patients with myeloproliferative neoplasms (133). However, another study showed that the treatment with ruxolitinib overcame resistance to cisplatin in *in vivo* and *in vitro* models of non-small-cell lung cancer (134). Since then, this compound has entered clinical trials in combination with other therapies for various forms of cancers, including chemotherapy for non-small-cell lung cancer (NCT02119650), refractory lymphoblastic leukemia (NCT02420717), refractory myeloid leukemia (NCT00674479), HER2 positive breast cancer (NCT02066532) and triple negative inflammatory breast cancer (NCT02876302) and in combination with the anti-PD1 drug pembroluzimab against stage IV triple negative breast cancer (NCT03012230). Some of these trials are still underway while results from others have not been revealed and many have even shown underwhelming results (135, 136). On the other hand, a JAK2 inhibitor SAR302503 has been shown to not only target therapy resistant lung cancers but also to abrogate PDL1 expression. Moreover, the sensitivity to this drug even correlated with higher expression of IRDS genes warranting further investigation in the clinic (137). Although most of these compounds do not exclusively target type I IFN signaling, their efficacy hints toward further exploration of novel drugs selectively targeting this pathway to overcome resistance.

A recent study highlighted the requirement to target negative regulators like the ISGs, SOCS1 and SOCS3 and identified a natural compound 6-hydroxy-3-*O*-methyl-kaempferol 6-*O*-glucopyranoside (K6G) which inhibited SOCS3 expression and stimulated type I IFN induced ISRE reporter expression (138). There is also strong evidence that USP18 is worth pursuing as a promising target and recent advances in solving its crystal structure along with ISG15 should help make this idea a reality in the clinic (139). On the other hand, an IRF inhibitor, LY294002, which targets IFN-β production via IRF3 inhibition (140), has been shown to sensitize cancer cells to chemotherapy in cervical cancer cells by enhancing mitochondrial JNK signaling. Agonists (141) and antagonists (142) of IFNAR are also under development and could prove useful against cancers. Besides, with the new reports of prolonged type I signaling associated with chronic inflammation in cancers, combining IFN inhibitors with other therapies might be beneficial, although this possibility remains to be experimentally demonstrated.

While oncolytic viruses represent another strategy to activate type I IFN signaling in the tumor microenvironment and are being tested alongside other therapies in multiple clinical trials (143), their efficacy has been shown to be enhanced by type I IFN pathway modulators. Ruxolitinib has been shown to inhibit the expression of ISGs like MDA5, RIG-I, MX1, IFIT3, and OAS1 and improve the infection of oncolytic HSV *in vitro* (144). A study by Esobar-Zarate et al. showed that IRF7, IRF9 and OAS1 but not MxA are upregulated in VSV resistant head and neck cancer cells and their treatment with ruxolitinib reduced IRF9 and IRF7 expression along with OAS1 expression and helps overcome resistance to this virus (145). This inhibitor has also been recently shown to overcome resistance to VSV in pancreatic ductal adenocarcinoma cells. In fact, adding polycations and ruxolitinib (which inhibits antiviral signaling) to VSV therapy successfully overcame the resistance of pancreatic carcinoma cells to VSV whilst also improving VSV attachment and replication (146). In another study, a histone deacetylase inhibitor, resminostat, was shown to improve the therapeutic effects of the measles vaccine virus by suppressing IFIT-1 function in hepatocellular carcinoma cells (147) A differential role for IFN-α and IFN-β was demonstrated in the induction of resistance of head and neck carcinoma cells to VSV. It was found that IFN-β, but not IFN-α, was crucial for maintaining persistent infection of these cells with VSV. When the cells were treated with antibodies against IFN-β, IFN-α or their combination before VSV infection, only anti-IFN-β protected cells from the infection significantly more than anti-IFN-α and the combination (148). These findings indicate that IFN-α is less effective at protecting cells from VSV oncolysis than is IFN-β, while both IFNs protect normal cells equivalently. These results could be instrumental in designing combinatorial therapies including OVs in the future.

## Conclusions

Type I IFN signaling is central to most anti-cancer therapies, new and the old alike. Since mutations in components of this pathway and chronic activation of the pathway both can be detrimental to the efficacy, assessing interferon signature genes before a specific therapy is initiated could be useful to tailor therapy. For example, the recently used IRDS scoring strategy identified breast and lung cancer patients with higher expression of ISGs as patients with poor responses to chemotherapy and radiotherapy (137). Indeed, even the use of IFN-only therapy might be detrimental for these type of patients. Strategies able to temporarily block IFN-signaling, preferably in cancer cells only, could be useful to limit chronic exposure to IFN and restore responsiveness to treatment.

On the other hand, as discussed above, blocking type I IFN signaling may render cancer cells resistant to other treatments, for example to anti-PD1 therapies, via downregulation of MHC class-I molecules (149). Therefore, inhibition of the blocking IFN pathway should be the therapeutic choice in accurately selected cases. The timing and duration of therapies aiming at blocking or activating type I IFN signaling are more relevant parameters to consider in the design of novel treatment schedules. The complexity of the involvement of type I IFNs in the interplay between cancer cells and TME requires further studies to more precisely identify suitable therapeutic targets in the various tumor settings. Moreover, in order to fine-tune combinatorial therapies, we need a better understanding of how type I IFN pathway interacts with other inflammatory pathways in the TME. There is also a need to understand exactly what various ISGs do in the TME—Is it just one ISG protein that is responsible for the therapeutic effects or does it have to be a signature that determines outcome in patients? Also, how do the functions of these ISGs change in the presence of therapy and do they contribute to stemness in that scenario?

Furthermore, even though various components of the type I IFN pathway are being targeted in the clinic, there is paucity of information on how these therapies affect downstream components of the IFN signaling the consequent counter-reactions in tumor cell signaling. The dynamic cross talk between tumor cells and the heterogeneous immune populations in different cancers adds a further level of complexity. Now that we are aware that chronic activation of type I IFN signaling may be causing “adaptive resistance” in many cancers, there is an even more urgent need to study these effects in more detail. In conclusion, reasons for failure of various anti-cancer therapies might lie under the basic questions around type I IFN signaling, its functions, cross-talk, mutations, timing and duration of exposure and it might be time to dig deeper into this puzzling scenario.

## Author contributions

RD and RM conceived the review. MB searched the literature and drafted the manuscript. RD and RM critically appraised the literature, wrote and approved final version of the manuscript.

### Conflict of interest statement

The authors declare that the research was conducted in the absence of any commercial or financial relationships that could be construed as a potential conflict of interest.
